# The histomolecular criteria established for adult anaplastic pilocytic astrocytoma are not applicable to the pediatric population

**DOI:** 10.1007/s00401-019-02088-8

**Published:** 2019-11-01

**Authors:** Albane Gareton, Arnault Tauziède-Espariat, Volodia Dangouloff-Ros, Alexandre Roux, Raphaël Saffroy, David Castel, Thomas Kergrohen, Fréderic Fina, Dominique Figarella-Branger, Mélanie Pagès, Franck Bourdeaut, François Doz, Stéphanie Puget, Christelle Dufour, Emmanuèle Lechapt, Fabrice Chrétien, Jacques Grill, Pascale Varlet

**Affiliations:** 1 Department of Neuropathology, GHU Paris-Neurosciences Sainte-Anne, 1 rue Cabanis, 75014 Paris, France; 2 Department of Pediatric Radiology, Necker-Enfants Malades Hospital, 149 Rue de Sèvres, 75015 Paris, France; 3 Department of Neurosurgery, GHU Paris-Neurosciences Sainte-Anne, 1 rue Cabanis, 75014 Paris, France; 4 Department of Biochemistry and Oncogenetics, Paul Brousse Hospital, 12 Avenue Paul Vaillant Couturier, 94804 Villejuif, France; 5 Nouvelles Thérapies Anticancéreuses, Unité Mixte de Recherche 8203 du Centre National de La Recherche Scientifique, Gustave Roussy Et Université Paris Saclay, Villejuif, France; 6 Aix-Marseille Univ, APHM, CNRS, INP, Inst Neurophysiopathol, CHU Timone, Service d’Anatomie Pathologique et de Neuropathologie, Marseille, France; 7 SIREDO Center (Care, Innovation and Research in Pediatric, Adolescent and Young Adults Oncology), Institut Curie and Paris University, Paris, France; 8 Department of Pediatric Neurosurgery, Necker-Enfants Malades Hospital, 149 Rue de Sèvres, 75015 Paris, France; 9 Department of Pediatric and Adolescent Oncology, Gustave Roussy, 114 Rue Edouard Vaillant, 94805 Villejuif, France

**Keywords:** Pilocytic astrocytoma with anaplastic features, Pediatric, DNA methylation profiling, MC-AAP, FGFR1, MAPK pathway

## Abstract

Pilocytic astrocytoma (PA) is the most common pediatric glioma, arising from a single driver MAPK pathway alteration. Classified as a grade I tumor according to the 2016 WHO classification, prognosis is excellent with a 10-year survival rate > 95% after surgery. However, rare cases present with anaplastic features, including an unexpected high mitotic/proliferative index, thus posing a diagnostic and therapeutic challenge. Based on small histomolecular series and case reports, such tumors arising at the time of diagnosis or recurrence have been designated by many names including pilocytic astrocytoma with anaplastic features (PAAF). Recent DNA methylation-profiling studies performed mainly on adult cases have revealed that PAAF exhibit a specific methylation signature, thus constituting a distinct methylation class from typical PA [methylation class anaplastic astrocytoma with piloid features—(MC-AAP)]. However, the diagnostic and prognostic significance of MC-AAP remains to be determined in children. We performed an integrative work on the largest pediatric cohort of PAAF, defined according to strict criteria: morphology compatible with the diagnosis of PA, with or without necrosis, ≥ 4 mitoses for 2.3 mm^2^, and MAPK pathway alteration. We subjected 31 tumors to clinical, imaging, morphological and molecular analyses, including DNA methylation profiling. We identified only one tumor belonging to the MC-AAP (3%), the others exhibiting a methylation profile typical for PA (77%), *IDH*-wild-type glioblastoma (7%), and diffuse leptomeningeal glioneuronal tumor (3%), while three cases (10%) did not match to a known DNA methylation class. No significant outcome differences were observed between PAAF with necrosis versus no necrosis (*p* = 0.07), or with 4–6 mitoses versus 7 or more mitoses (*p* = 0.857). Our findings argue that the diagnostic histomolecular criteria established for anaplasia in adult PA are not of diagnostic or prognostic value in a pediatric setting. Further extensive and comprehensive integrative studies are necessary to accurately define this exceptional entity in children.

**Electronic supplementary material:**

The online version of this article (10.1007/s00401-019-02088-8) contains supplementary material, which is available to authorized users.

## Introduction

Pilocytic astrocytoma (PA) is the most common pediatric brain tumor (17.6% of childhood primary brain tumors), with peak incidence between 5 and 15 years [19]. These slow growing tumors are designated as grade I by the World Health Organization (WHO) classification of tumors of the central nervous system (CNS) and have a generally favorable outcome, with a 10-year survival rate of 95% [19]. Patient age and extent of resection represent key prognostic factors [30]. Molecular alterations of the mitogen-activated protein kinase (MAPK) pathway are characteristics of PA, the most frequent being a fusion of the *BRAF* gene with *KIAA1549* in 66% of cases, resulting from a tandem duplication at chromosome 7p34 [12]. Other alterations of MAPK have been observed, such as mutations of *BRAF, FGFR1*, *KRAS*, *PTPN11,* and *NF1* genes, and fusions of *NTRK2* and *FGFR1* [11, 19]. Aside from this single genetic alteration which drives oncogenesis, most PAs have a relatively stable karyotype, or focal gains of chromosomes 5 or 7 [16].

Histopathologically, tumors with conventional PA morphology with signs of histopathological malignancy (mitoses, necrosis, and increased cellularity) have been designated in the literature with variable terminology, such as atypical PA, malignant PA, PA with anaplasia, PA with anaplastic features (PAAF), or anaplastic pilocytic astrocytoma (ANA PA) [19, 27, 29]. The 2016 WHO classification uses the term PA with anaplasia, but for this study we have chosen to use PAAF to discriminate the histomolecularly defined subgroup from epigenetically defined MC-AAP. These tumors may arise either de novo or in the context of malignant transformation. The WHO classification specifies brisk mitotic activity (≥ 4 mitoses per 10 high power fields (HPF)) with or without necrosis, as criteria to look for in PAAF [19, 29]. WHO grade is still to be determined. Nonetheless, this diagnosis remains difficult, as these morphological features are still compatible with the diagnoses of classical PA, grade I, with degenerative, necrotic or hemorrhagic modifications, or even glioblastoma, grade IV.

Recently, large-scale epigenetic analyses mapping methylated CpG sites on tumoral genomes have allowed for the characterization of genetically defined tumoral subtypes in the CNS [3]. Developments in DNA methylation profiling as a classifying tool have described MC-AAP as a new, distinct genetic subclass from conventional PA [DNA methylation classes: PA posterior fossa (PF), PA midline (MID), PA supratentorial (SUP)] [3, 26]. It is important to note that this new DNA methylation class was described mostly in adults. Indeed, only six pediatric patients were included in this large patient cohort [26]. In contrast to classical PA, supplementary alterations of *CDKN2A/B* and loss of ATRX protein expression were found in the MC AAP in addition to the classical single MAPK alteration.

The newfound interest in this histomolecular entity raises the question of its relevance in a pediatric setting. Indeed, of the all PAAF cases described in the literature, only 22 cases were pediatric, with or without MAPK alteration [17, 20, 21, 24, 27, 28] including 6 with DNA methylation profiling [26]. Our study consists of a retrospective analysis of 31 pediatric (< 18 years) PAAF cases defined according to strict histomolecular criteria. We integrated clinical, radiological, histopathological, immunohistochemical, molecular, DNA methylation profiling, and copy number profile (CNP) data to better characterize these tumors in children.

## Materials and methods

### Cohort selection

**Fig. 1:**
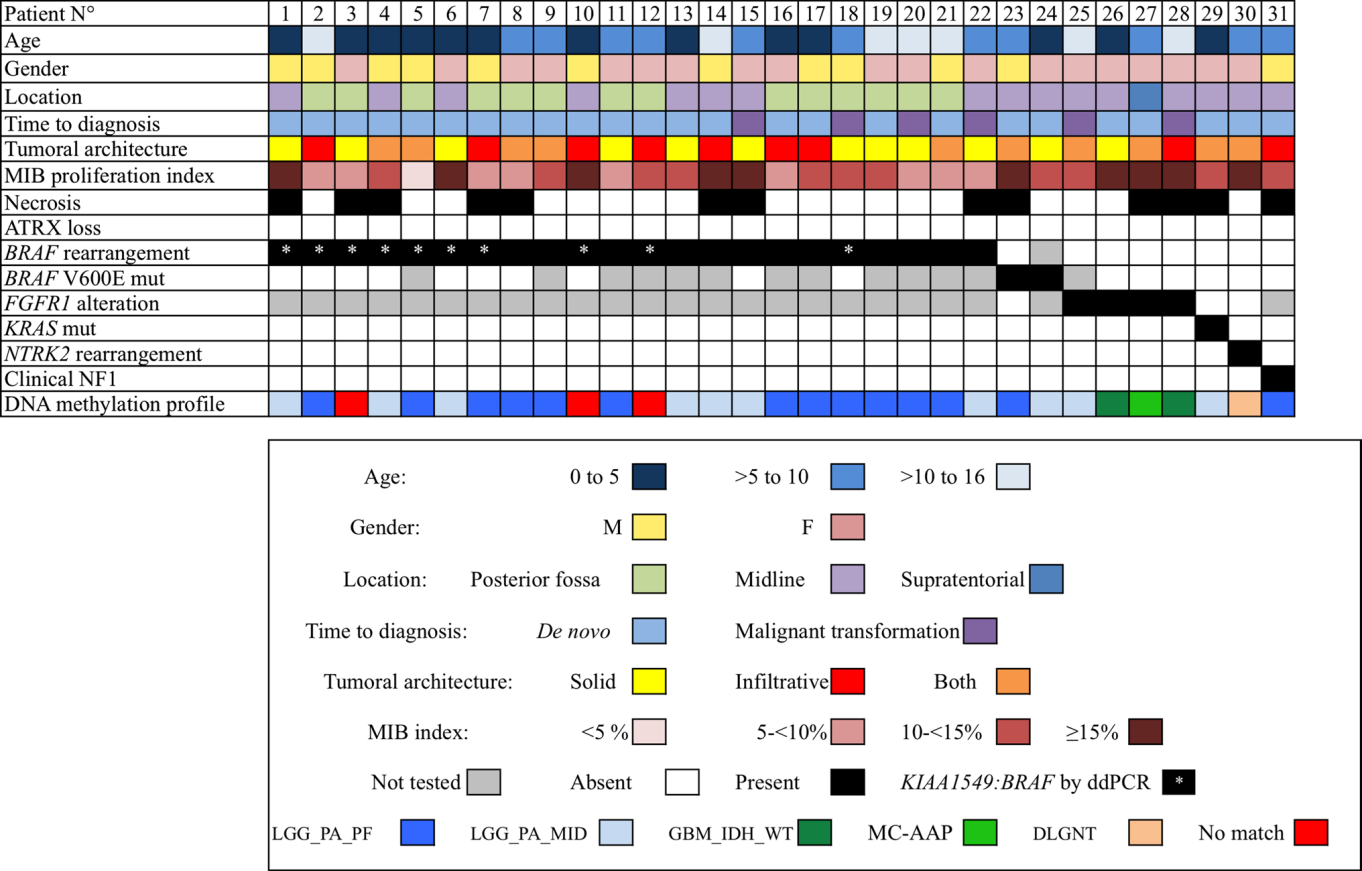
Clinical, Radiological, Histopathological, and Molecular Characterization of 31 pediatric PAAF

This study was approved by the GHU Paris-Neurosciences Sainte-Anne’s local ethics committee (INDS: MR 1409210519). We selected 31 pediatric cases that received the institutional diagnosis of PAAF at the Necker-Enfants Malades Hospital and the GHU Paris-Neurosciences Sainte-Anne between 2008 and 2019, out of 511 classical pediatric PA diagnosed during this time period. Selected tumors satisfied these three inclusion criteria: (i) classical morphology of PA, (ii) MAPK alteration, and (iii) at least 4 mitoses per 10 HPF (2.3 mm^2^), with or without necrosis [19]. Since no specific reference area has been published, we selected 2.3 mm^2^, as used for pleomorphic xanthoastrocytoma and choroid plexus tumors [19]. These criteria were present either de novo, or in the context of malignant transformation of PA at recurrence. These data are summarized in Fig. 1.

### Clinical and radiological data

Clinical data were collected from Gustave Roussy Institute, Curie Institute, and Necker-Enfants Malades Hospital. Complete clinical history was retrieved when possible: gender, age at diagnosis, family history, known tumor predisposition syndrome, symptoms at diagnosis, extent of resection, neoadjuvant or adjuvant treatments (chemotherapy, radiation), progression-free survival (PFS) defined as the date of the first radiologically confirmed relapse, long-term follow-up, and overall survival (OS). Extent of resection was noted as gross total resection (GTR), subtotal resection (STR), defined as the resection of more than 90% of the tumor volume, or partial resection (PR) (less than 90% of tumor volume). All cases were subject to local radiological review by an experienced pediatric neuroradiologist (VDR). Because of the long inclusion time period, imaging protocol varied among patients. Computed tomography (CT) scan, when available, was used to assess tumor density, calcifications, and bleeding. MRI protocol always included T1-weighted, T2-weighted, and post-contrast T1-weighted images, and inconsistently FLAIR, diffusion-weighted images, and pre-contrast Arterial Spin Labeling (ASL) perfusion-weighted images. To assess characteristic imaging features of PAAF, this cohort was compared to a previously published posterior fossa classical PA cohort and to an unpublished cohort of optic pathway gliomas [6].

### Histopathological examination

Formalin-fixed paraffin-embedded (FFPE) tissue samples for each patient were retrieved from the GHU Paris-Neurosciences Sainte-Anne archives. Haematoxylin–Phloxine–Saffron (HPS)-stained slides for all cases underwent central review by two experienced neuropathologists (PV, ATE) to confirm morphological diagnoses as well as mitotic count for 2.3 mm^2^. Necrosis, microvascular proliferation, Rosenthal fibers, eosinophilic granular bodies, calcifications, and lymphocyte infiltration were noted as present or absent. Tumoral architecture was labeled biphasic, oligodendroglial-like, or piloid. The infiltration pattern was assessed and tumors were described as solid, infiltrative, or both.

### Immunohistochemistry

Immunohistochemical analyses were carried out with antibodies specific for ATRX (*n* = 31) (Clone BSB108, 1:200; Bio SB, Santa Barbara, USA), MIB-1 labeling index (*n* = 31) (Clone M7240, 1:200; Aligent-Dako, Les Ulis, France), monoclonal mouse anti-human PTEN (*n* = 31) (Clone 6H2.1, 1:100; Dako, Les Ulis, France), Phospho-S6 (*n* = 31) (Ser235/236, 1:400; Cell Signaling Technology Inc, Leiden, The Netherlands), H3K27me3 (*n* = 31) (Clone A0821D, 1:1250; Diagenode, Seraing, Belgium), and BRAFV600E (*n* = 18) (Clone VE1, 1:100; Spring Biosciences, Pleasanton, USA) on the Benchmark XT immunostainer.

### Fluorescent in situ hybridization (FISH) and molecular analyses

In order of frequency: FISH analyses for *BRAF* gene rearrangements using the ZytoLight SPEC *BRAF* dual color break apart probe (Zytovision, Bremerhaven, Germany) were carried out in all cases. Fluorescent signals were counted in 100 tumoral nuclei with the DM600 Leica fluorescent microscope (Leica Biosystems, Richmond, IL). If negative, rearrangements of NTRK2 were searched for using the ZytoLight SPEC *NTRK2* dual color break apart probe (Zytovision, Bremerhaven, Germany). When negative, a brain tumor-specific sequencing panel was developed using Massarray iPlex technology and Massarray online design tools (Agena Biosciences) to detect mutations in *FGFR1*, and *BRAF* genes, as previously described [23]. Next-generation sequencing (NGS) was also performed in rare cases according to the Illumina NextSeq 500 protocol (Illumina, San Digeo, CA, USA), which allowed us to detect mutations of *KRAS, NRAS* and *HRAS*. When necessary, droplet digital polymerase chain reaction (ddPCR) was performed to confirm *KIAA1549:BRAF* fusion or to search for *FGFR1* duplications, as previously described [1].

FISH for *PTEN* was performed on all samples using the Vysis *PTEN*/CEP10 FISH probe kit (Abbott Molecular Inc., Des Plaines, IL, USA). FISH for 1p19q, using the Vysis 1p36/1q25 and 19q13/19p13 FISH probe kit (Abbott Molecular Inc., Des Plaines, IL, USA) was performed in all spinal cases. FISH for *CDKN2A* was performed using the Vysis *CDKN2A*/CEP 9 FISH probe kit (Abbott Molecular Inc., Des Plaines, IL, USA) after DNA methylation profiling, to confirm alterations observed on the CNP. Fluorescent signals were counted in 100 tumoral nuclei with the DM600 Leica fluorescent microscope (Leica Biosystems, Richmond, IL, USA).

### RNA sequencing and analysis

RNAseq was performed on two cases on the Integragen platform (Evry, France). Libraries were prepared with TruSeq Stranded mRNA kit according to the supplier recommendations. Briefly, the key stages of this protocol were, successively, the purification of PolyA containing mRNA molecules using poly-T oligo attached magnetic beads from 1 µg total RNA, a fragmentation using divalent cations under elevated temperature to obtain approximately 300 bp fragments, double-stranded cDNA synthesis and finally Illumina adapters ligation and cDNA library amplification by PCR for sequencing. Sequencing was then carried out on paired-end 75 bb using Illumina NextSeq500.

The software used for RNA alignment was TopHat2.1.0, including Bowtie2. A quantitative expression profile is generated using Cufflinks 2.2.0 [31].

Single nucleotide variants (SNV) and small insertions and deletions (Indels) were detected with Samtools/BcfTools (Broad Institute). Functional consequences of variants on genes, transcripts, and protein sequence, as well as regulatory regions, were predicted by Variant Effect Predictor (VEP release 83) (stop, splicing, missense, synonymous), as well as by location of the variants (e.g., upstream of a transcript, in coding sequence, in non–coding RNA, in regulatory regions). Regarding missense changes, two bioinformatics predictions for pathogenicity were available SIFT (sift5.2.2), PolyPhen (2.2.2). Other information like quality score, homozygote/heterozygote status, count of variant allele reads, and the presence of the variant in the COSMIC database (version 71) was reported.

### DNA methylation profiling and t-SNE analysis

DNA extraction from FFPE was performed using the QIAamp DNA FFPE Tissue Kit and the Qiacube (QIAGEN, Hilden, Germany) according to the manufacturer’s instructions. 250–500 ng of DNA was extracted from each tissue sample. Kits used for bisulfite conversion and reparation were the Zymo EZ DNA methylation kit and ZR-96 DNA Clean and Concentrator-5 (Zymo Research, Irvine, CA, USA) and bisulfite DNA was processed using the Illumina Infinium HD FFPE DNA Restore kit and Infinium FFPE QC kit (Illumina, San Diego, CA, USA).

The DNA was then processed using the Illumina Infinium HumanMethylation EPIC Bead-Chip array (Illumina, San Diego, CA, USA) according to the manufacturer’s instructions. The iScan control software was used to generate raw data files from the BeadChip in IDAT format and analyzed using GenomeStudio version 2.0 (Illumina, San Diego, CA, USA). The following filtering criteria were applied: removal of probes targeting the X and Y chromosomes, removal of probes containing single-nucleotide polymorphisms (dbSNP132 Common) within five base pairs of and including the targeted CpG site, and removal of probes not mapping uniquely to the human reference genome (hg19), allowing for one mismatch [3]. The raw IDAT files were uploaded to https://www.molecularneuropathology.org for supervised analysis using the Random Forest methylation class prediction algorithm, as previously described [3]. Copy number profiles were calculated as previously described [3].

Raw signal intensities were obtained from iDat files using the minfi Bioconductor package v1.28.4. Background correction and dye-bias correction were performed on each sample. After a correction for the type of material tissue (FFPE or frozen) was performed with the removeBatchEffect function (limma package v3.38.3). Filtering criteria of probes were removal of probes targeting X or Y chromosomes, removal of probes containing single nucleotide polymorphisms, and probes not included in the EPIC array. We selected the most variable probes for t-SNE (t-Distributed Stochastic Neighbor Embedding) (SD > 0.30) with parameter theta = 0, pca = TRUE, max_iter = 2500, perplexity = 30, based on the method by Capper et al. [3]. t-SNE was performed using the Rtsne package, version 0.15. The following glioma reference classes were included: MC-AAP (here annotated as ANA_PA; 21 cases); diffuse leptomeningeal glioneuronal tumor (DLGNT; 8 cases); GBM, G34 mutant (GBM_G34; 41 cases); GBM, mesenchymal subtype (GBM_MES; 56 cases); GBM of the midline (GBM_MID; 24 cases); GBM, MYCN mutant (GBM_MYCN; 16 cases); GBM, RTK I subtype (GBM_RTK_I; 64 cases); GBM, RTK II subtype (GBM_RTK_II; 143 cases); GBM, RTK III subtype (GBM_RTK_III; 13 cases); ganglioglioma (LGG_GG; 21 cases); PA or ganglioglioma of the supratentorial hemispheres (LGG_PA_GG_ST; 24 cases); PA of the midline (LGG_PA_MID; 38 cases); PA of the posterior fossa (LGG_PA_FP; 114 cases). Detailed descriptions of the reference methylation classes are available on https://www.molecularneuropathology.org

### Statistical analyses

Univariate analyses were carried out using chi-square or Fisher’s exact test for comparing categorical variables, and the unpaired *t* test or Mann–Whitney rank-sum test for continuous variables, as appropriate. The Kaplan–Meier method, using log rank tests to assess significance for group comparisons, plotted unadjusted survival curves for PFS and OS. A Cox proportional hazards model was performed in a multivariate analysis. We created Cox proportional hazards regression models on the whole series using a backward stepwise approach, adjusting for predictors previously associated at the *p* < 0.2 level with recurrence in unadjusted analysis. A probability value < 0.05 was considered statistically significant. Statistical analyses were performed using the JMP software (version 14.1.0, SAS Institute Inc.).

## Results

### Clinical and radiological characteristics

The median and mean patient age were 7.0 and 6.8 years, respectively, with a range 3 months to 15.9 years. The male to female ratio showed a slight female predominance (M:F ratio 0.63). Of the 31 patients, 25 (81%) presented with PAAF de novo, whereas 6 cases (19%) originated from the re-resection of a previously known PA. In these 6 cases, the initial diagnosis of classical PA without anaplasia was reexamined and confirmed. Eighteen (58%) patients had gross total resection (GTR) (*n* = 9) or subtotal resection (STR) (*n* = 9). Thirteen (42%) patients had partial resection (PR). Patients were treated by observation only (*n* = 16), adjuvant or neo-adjuvant chemotherapy (*n* = 12), irradiation only (*n* = 1), or radiation and chemotherapy (*n* = 2) (Table 1).

**Table 1:** Clinical characteristics of 31 pediatric patients with PAAF

Case	Age	Sex	Tumor location	APA on first resection or other resection	Resection type	Adjuvant treatment	Recurrence interval (months)	Status at last follow-up	OS (months)
1	1.9	M	Optic chiasm	De novo	STR	CT	9.6	A	27.4
2	13.0	M	Right cerebellar hemisphere	De novo	GTR	None	None	A	3.7
3	4.2	F	Cerebellar hemispherse	De novo	STR	None	10.8	A	38.3
4	0.4	M	Hypothalamus	De novo	PR	CT	2.4	A	71.3
5	3.3	M	Left cerebellar hemisphere	De novo	GTR	None	None	A	15.6
6	0.7	F	Hypothalamus	De novo	PR	CT	2.1	A	86.1
7	1.0	M	Right cerebellar hemisphere	De novo	STR	None	3.1	A	39.4
8	10.0	F	Left cerebellar hemisphere	De novo	GTR	None	None	A	4.1
9	5.5	F	Right cerebellar hemisphere	De novo	GTR	None	None	A	6.8
10	0.4	M	Optic chiasm	De novo	PR	CT	8.2	DOD	27.5
11	10.1	F	Cerebellar vermis	De novo	STR	None	None	A	24.6
12	5.3	F	Left cerebellar hemisphere	De novo	GTR	None	None	A	13.3
13	0.5	F	Hypothalamus	De novo	PR	CT	3.4	DOD	46.7
14	15.0	M	Hypothalamus	De novo	PR	None	None	A	27.6
15	7.8	F	Optic chiasm	Malignant transformation	STR	CT + RT	29.4	A	103.8
16	3.3	F	Cerebellar vermis	De novo	STR	None	1.0	A	6.5
17	1.7	M	Right cerebellar hemisphere	De novo	STR	None	None	A	8.8
18	9.8	M	Left cerebellar peduncle	Malignant transformation	PR	CT	11.9	A	51.1
19	12.7	F	Right cerebellar hemisphere	De novo	GTR	None	39.4	A	53.5
20	15.2	F	Right cerebellar hemisphere	Malignant transformation	GTR	None	None	A	49.7
21	13.0	M	Vermis and left cerebellar hemisphere	De novo	STR	None	None	A	2.5
22	8.8	F	Hypothalamus	Malignant transformation	PR	CT	None	A	1.4
23	7.5	M	3rd ventricle	De novo	PR	CT	13.8	A	13.7
24	0.6	F	Hypothalamus	De novo	PR	none	None	DOS	0.0
25	15.4	F	Hypothalamus	Malignant transformation	PR	RT	11.5	A	37.7
26	0.8	F	Spinal	De novo	PR	CT	1.2	A	12.9
27	8.0	F	Right parietal	De novo	GTR	CT + RT	13.4	DOD	37.0
28	15.9	F	Hypothalamus	Malignant transformation	STR	CT	6.9	DOD	8.2
29	1.6	F	Hypothalamus	De novo	PR	CT	1.0	A	18.9
30	7.0	F	Spinal	De novo	PR	None	None	A	1.7
31	8.8	M	Bulbo-medullary junction	De novo	GTR	CT	2.5	A	12.2

*A* alive, *CT* chemotherapy, *DOD* dead of disease, *DOS* dead of surgery, *F* female, *GTR* gross total resection, *M* male, *PR* partial resection, *RT* radiotherapy, *STR* subtotal resection

Tumor location was subdivided into posterior fossa (*n* = 14, cerebellar hemispheres *n* = 11; cerebellar vermis *n* = 3), midline (*n* = 16, including hypothalamus/optic chiasm (*n* = 12), the spinal cord (*n* = 3) and 3rd ventricle (*n* = 1)), and supratentorial hemispheric (*n* = 1) (Table 1). Imaging studies revealed radiological features close to classical PA, except for perfusion values. Fourteen patients had available CT scans, showing a hypodense tissular component in all cases and 3/14 had calcifications. On MRI, all tumors were well delineated with clearly marked borders, with peri-tumoral edema on T2-weighted images in 48% (n = 15/31). Contrast enhancement was always present but with variable intensity. Apparent diffusion coefficient was high in 20/24 tested tumors and was intermediate in 3 cases.

Posterior fossa PAAF showed no specific features in comparison to classical PA: they were hemispheric cerebellar tumors with a cystic component and post-contrast enhancement, revealing a mural nodule (*n* = 12/14) or cyst wall enhancement (*n* = 2/14). Hypothalamic/Optic chiasm PAAF were always large tumors, ranging from 3 to 8 cm, and mostly hypothalamic, with intense post-contrast enhancement.

Seventeen patients had available ASL cerebral blood flow (CBF) values, ranging from 28 to 204 mL/min/100 g, with a median value of 56 mL/min/100 g (IQR [48–75], higher than classical PA (median 36 ml/min/100 g, IQR [28–48], *p* < 0.001) [6]. ASL CBF was higher than in control groups (posterior fossa PA with anaplasia median 49 ml/min/100 g vs 34 ml/min/100 g for classical PA (*p* = 0.008); optic pathways PA with anaplasia median 66 ml/min/100 g vs 39 ml/min/100 g for classical PA (*p* = 0.04)).

Eight patients (26%) presented with intracranial metastatic lesions at the time of discovery. When spinal MRI was available (n = 11), 36% (4/11) had metastatic lesions.

### Histopathological and immunohistochemical characterization

All cases presented with typical PA morphology. Mitotic count ranged from 4 to 32 per 2.3 mm^2^ (mean 7, median 7). The MIB-1 proliferation index ranged from 3 to 50% (mean 14%, median 11%). ATRX expression was maintained in all 31 cases. In all cases, H3K27me3 was preserved, allowing us to exclude potential tumors with combined MAPK alteration and H3K27M mutation [22]. Phospho-S6 staining was strongly positive in all cases except one with weak staining (patient 26). Immunohistochemistry for PTEN was interpretable in 29/31 cases and was variable, with conserved expression in 20 cases, partial loss in 5 cases, and complete loss of expression in 4 cases (patients 10, 26, 27, and 30). All morphological and immunohistochemical features are detailed in supplementary Table 1, online resource.

### Genomic DNA methylation

850 K DNA methylation profiling was performed on all 31 PAAFs. Three cases did not correspond to a specific DNA methylation class and were subsequently designated as “no match cases” (patients 3, 10, and 12); since they yielded unreliably low classifier scores below 0.18 (supplementary Table 1, online resource). They did, however, cluster as PA in t-SNE analysis (case 10 to LGG_PA_MID, cases 3 and 12 to LGG_PA_PF). Twenty cases received a calibrated score superior to 0.90. Seven tumors yielded a classifier diagnosis below the established threshold of 0.90 (0.89–0.46); however, in all these cases the highest calibrated score was consistent with clinical, radiological, histomolecular data and t-SNE cluster. In one medullar case, the highest calibrated score obtained was 0.32 for GBM_IDH_WT_MID (patient 26).

Excluding the “no match cases”, 24 (86%) cases were classified as pilocytic astrocytoma (LGG_PA_PF *n* = 14; LGG_PA_MID *n* = 10) and from here onwards will be designated as classical PA. One case was classed as diffuse leptomeningeal glioneuronal tumor (DLGNT) (patient 30), and two as glioblastoma, *IDH1/2* wild type (GBM_IDH_WT) (patients 26 and 28). Only one case received the DNA methylation-based diagnosis of MC-AAP (patient 27).

**Fig. 2:**
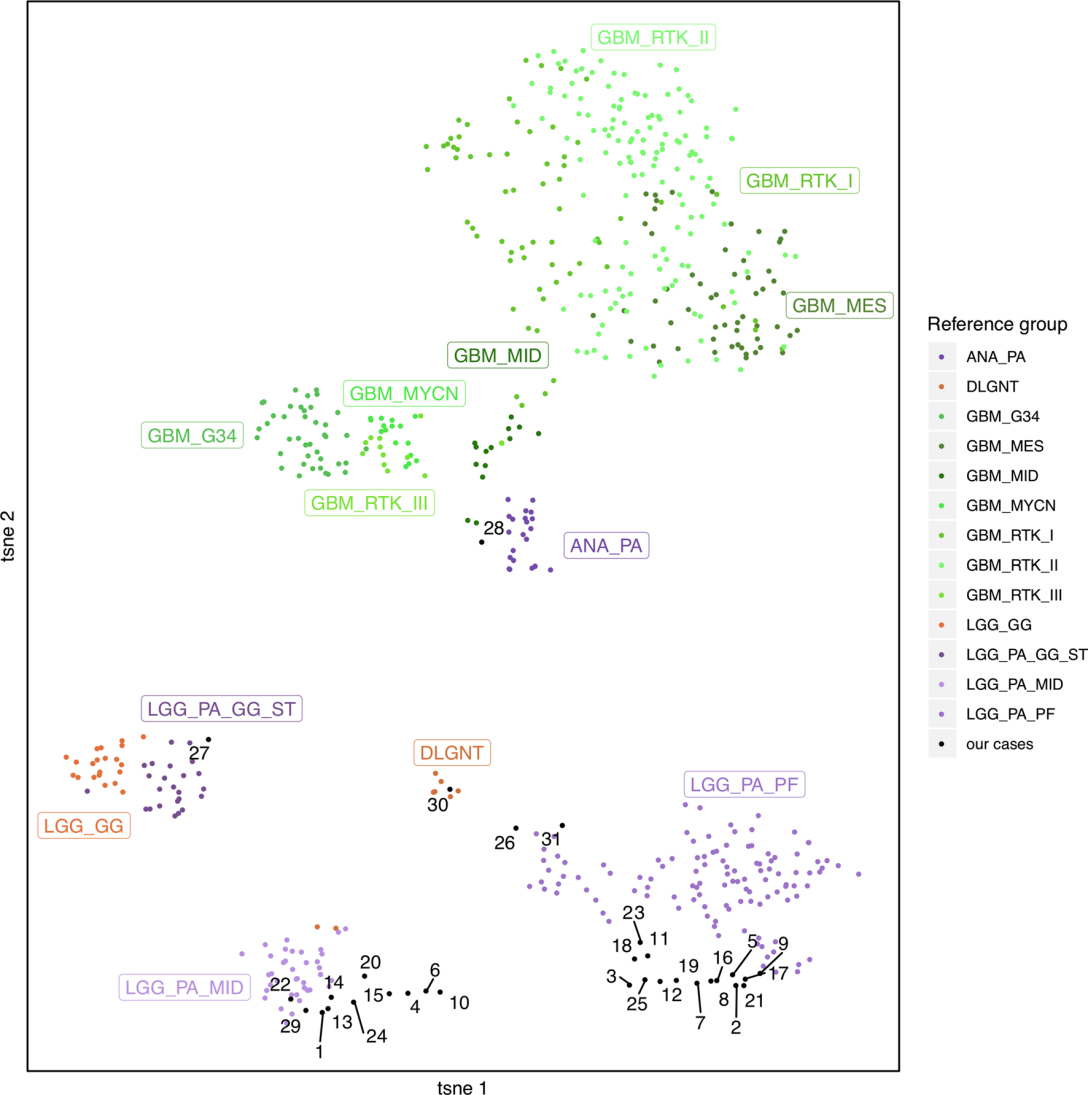
t-SNE plot of the 31 PAAF cases. Reference classes: *ANA_PA* anaplastic pilocytic astrocytoma, *DLGNT* diffuse leptomeningeal glioneuronal tumor, *GBM_G34* glioblastoma with G34 mutation, *GBM_MES* glioblastoma, mesenchymal subtype, *GBM_MID* glioblastoma of the midline, *GBM_MYCN* glioblastoma, MYCN mutant GBM_RTK_1: glioblastoma, RTK I subtype, *GBM_RTK_II* glioblastoma, RTK II subtype, *GBM_RTK_III* glioblastoma, RTK III subtype, *LGG_GG* ganglioglioma, *LGG_PA_GG_ST* PA or ganglioglioma of the supratentorial hemispheres, *LGG_PA_MID* PA of the midline, *LGG_PA_FP* PA of the posterior fossa

The results from DNA methylation profiling and t-SNE analysis (cluster analyses with 729 reference cases from 13 distinct MCs) were concordant in 29/31 samples (27 classical PA, one DLGNT, and one GBM_MID), as seen in Fig. 2.

**Fig. 3:**
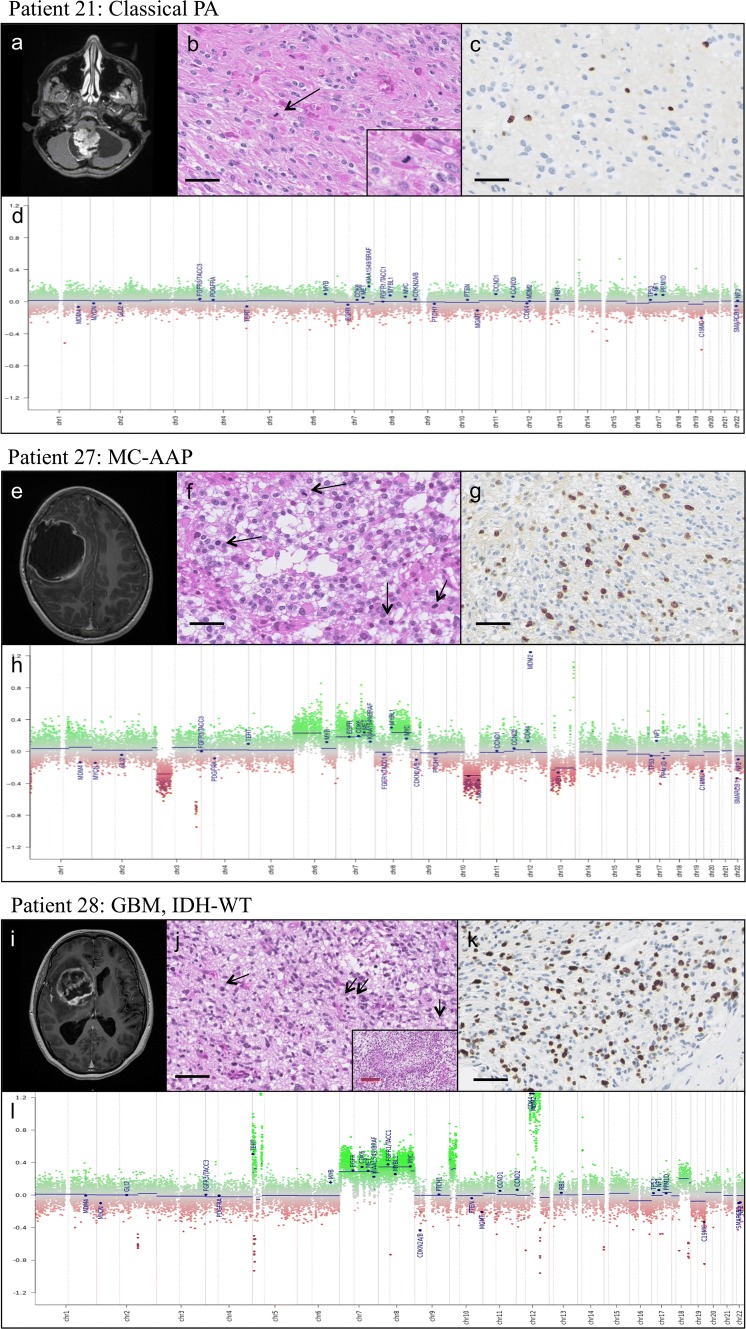
Comparative radiological, histological, molecular and CNP characteristics of PA (patient 21), MC-AAP (patient 27), and GBM (patient 28). Patient 21 Classical PA: **a** MRI: 3D T1 with gadolinium injection, showing a classical cerebellar PA features with both cystic and nodular portions and strong contrast enhancement. **b** Histological features: biphasic architecture, Rosenthal fibers, eosinophilic granular bodies, and mitosis (see insert and arrowhead), **c** MIB1 labeling index estimated at 10%, **d** flat CNP characteristic of classical PA. Patient 27 MC-AAP: **e** MRI: 3D T1 with gadolinium injection showing a right parietal mostly cystic lesion with a thick, irregular Contrast enhancing border. **f** Histological features: oligodendroglial-like morphology, numerous mitoses (arrowheads). No Rosenthal fiber or eosinophilic granular bodies were observed, **g** MIB1 labeling index estimated at 30%, **h** CNP with many chromosomal gains and losses, as well as focal amplifications, including MDM2. **Patient 28 GBM, IDH-WT:i** MRI: 3D T1 with gadolinium injection showing a right mass growing from the optic chiasm into the basal ganglia. **j** Histological features: biphasic morphology with numerous mitoses (arrowheads) and palisading necrosis (see insert), **k** MIB1 labeling index estimated at 40%, **l** CNP showing multiple chromosomal gains and losses, including a loss of *CDKN2A/B*, and focal amplifications of MDM2 and CDK4. Black scale bar corresponds to 50 μm, red scale bar corresponds to 200 μm

Two cases exhibited discordant grouping between hierarchical clustering and t-SNE analysis. For the medullar tumor (patient 26), the highest calibrated score was 0.32 for GBM_MID but clustered in t-SNE close to LGG_PA_PF. It is, however, interesting to note that the calibrated score for LGG_PA_PF was 0.25. For patient 27, the main calibrated score was MC-AAP (0.89) but in t-SNE analyses, clustering was near LGG_PA_GG_ST. In this case, however, the calibrated score for LGG_PA_GG_ST was low, estimated as 0.013.

These discordances can be explained by differences in methods in variable selection and reference subgroups. Our analysis used a reduced number of sample references compared to the classifier, which can lead to a different t-SNE profile.

### Copy number profiles (CNP)

To further characterize this entity, we analyzed chromosomal copy number variants from the CNPs. Of the tumors which received the DNA methylation classifier-based diagnosis of PA, 14/24 had flat CNPs with the characteristic focal low level 7q gain indicating the *BRAF* fusion. Four out of 24 showed characteristic gains of chromosomes 5 and/or 7, as it has been previously described in PA [16]. One case showed only a gain of chromosome 8. Interestingly, the 5 remaining PAs had complex chromosomal rearrangements, with focal gains of chromosomes 5, 6, 7, 8, 10, 11, 12, 18, and/or 20. One of the complex cases showed a focal deletion on chromosome 9 at the *CDKN2A* locus. The DLGNT and one GBM, *IDH*-WT, had relatively flat profiles, while the other GBM, *IDH*-WT had a very complex CNP with a characteristic gain of chromosome 7. The MC-AAP had a very complex CNP, with deletions of chromosomes 3p, 10q, and 13q, gains of chromosomes 6, 7, and 8q.

### Orthogonal validation of DNA methylation groups

DNA methylation profiling was concordant with MRI location in 26/28 conclusive cases. 12/12 cerebellar tumors were classified as LGG_PA_PF by DNA methylation profiling. 10/15 midline PAs (including the optic chiasm and spine) were classified as LGG_PA_MID by DNA methylation profiling. Two discrepancies arose: (1) patient 23 had a tumor which arose in the third ventricle from the ventral border of the tectal plate (reevaluated by an expert neuro-radiologist), and received a score for posterior fossa PA more significant than for midline PA (PF calibrated score 0.67, midline calibrated score 0.19, with a calibrated score for PA of 0.88), (2) patient 31’s tumor was predominantly medullary with a small bulbar component, but classified by the DNA methylation profiling as posterior fossa PA. The three remaining cases were also midline: either spinal (the DLGNT and one GBM, *IDH*-WT) or hypothalamic (GBM, *IDH*-WT). The MC-AAP was supratentorial.

Figure 3 comparatively illustrates the different radiological, histopathological, molecular and CNP characteristics of patients 21 (LGG_PA_PF), 27 (MC-AAP), and 28 (GBM, IDH-WT)

All tumors that underwent methylation profiling with a diagnosis of PA had a radiological and histopathological diagnosis compatible with classical PA, as illustrated in Fig. 3a–d. Patient 21’s tumor received the DNA methylation-based diagnosis of classical PA. MRI showed classical cerebellar PA features with both cystic and nodular portions and strong contrast enhancement (Fig. 3a). The biphasic tumor presented with Rosenthal fibers, eosinophilic granular bodies, and few mitoses (7/10HPF) (Fig. 3b), and MIB1 labeling index was estimated at 10% (Fig. 3c). The flat CNP was characteristic of classical PA (Fig. 3d).

The tumor from patient 27 received the DNA methylation profile of MC-AAP. Patient 27 was an 8-year-old girl who presented with headaches and paresis of the left arm. MRI revealed a cystic right parietal mass with contrast enhancement along the cyst border (Fig. 3e). This biphasic tumor presented with a very elevated mitotic count (17 mitoses for 2.3 mm^2^), necrosis, and microvascular proliferation (Fig. 3f) The MIB1 labeling index was estimated at 30% (Fig. 3g**)**. The CNP revealed an amplification of *MDM2* gene which was associated with an overexpression of the protein MDM2 by immunohistochemistry (MDM2 (*n* = 1) [Clone IF2, 1:100; Thermofisher, Waltham, USA)] and a subtle loss of chromosome 10q, where FISH for *PTEN* revealed a monosomy (Fig. 3h). It harbored *FGFR1* K678E and RAD50 R365Q pathogenic variants detected by RNA sequencing.

The tumor from patient 28 received the DNA methylation-based diagnosis GBM, *IDH*-WT. Patient 28 was a 15-year-old girl who presented with headaches and intra-cranial hypertension. MRI revealed a cystic hypothalamo-chiasmatic lesion with contrast enhancement along the cyst border (Fig. 3i). The infiltrative, biphasic tumor had Rosenthal fibers, eosinophilic granular bodies, 32 mitoses for 2.3 mm^2^, palisading necrosis, and microvascular proliferation (Fig. 3j**)**. The MIB1 labeling index was estimated at 40% (Fig. 3k). The CNP revealed an amplification of *MDM2* and *CDK4* genes, as well as a loss of *CDKN2A/B*, frequently described in GBM (Fig. 3l)*.* This tumor harbored both an *FGFR1* in frame insertion and a *PIK3CA* E454K mutation detected by RNA sequencing.

The tumor from patient 26 received the DNA methylation-based diagnosis of GBM, *IDH*-WT with a very low score of 0.32, but clustered to PA in t-SNE. Patient 26 was an 8-month-old girl who presented with hemiparesis. MRI revealed a spinal lesion ranging from C4–T8, with necrosis and contrast enhancement along the cyst border. This oligo-like tumor displayed anaplastic features such as a high mitotic index (16 mitoses for 2.3 mm^2^) and microvascular proliferation, but no necrosis. The tumor harbored a double *FGFR1* mutation (N546S and K656E) detected by sequencing.

The tumor from patient 30 received the DNA methylation-based diagnosis of DLGNT. Patient 30 was a 7-year-old girl who presented with headaches and neck pain. On MRI, she had a large purely intramedullary spinal lesion ranging from the medulla oblongata to T7, with strong contrast enhancement and necrotic parts. This oligo-like tumor presented with microvascular proliferation, without necrosis, and 6 mitoses for 2.3 mm^2^, and harbored an *NTRK2* rearrangement by FISH [ZytoLight SPEC *NTRK2* dual color break apart probe (Zytovision, Bremerhaven, Germany)]. Interestingly, 1p FISH was difficult to interpret when first performed. This tumor was classified by DNA methylation as DLGNT (calibrated score 0.99), with a subtle loss of chromosome 1 on CNP. Therefore, 1p FISH was redone on multiple tumor fragments and, after reanalysis, was interpreted as a partial deletion due to probable intra-tumoral heterogeneity (Supplementary Fig. 1, online resource). Reexamination of the initial imaging found no leptomeningeal dissemination of the tumor (no MRI follow-up available). Based on the spinal location, *NTRK2* rearrangement, and heterogeneous 1p loss in tumor cells, we retained the diagnosis of DLGNT.

In all “no match cases”, FISH for *BRAF* showed a typical rearrangement. The rearrangement pattern evoked a duplication as frequently seen in the case of a *KIAA1549:BRAF* fusion. A sensitive secondary technique (ddPCR) confirmed the *KIAA1549:BRAF* fusion in all three cases. Therefore, the diagnosis of classical PA was retained.

### Outcome analysis

**Fig. 4:**
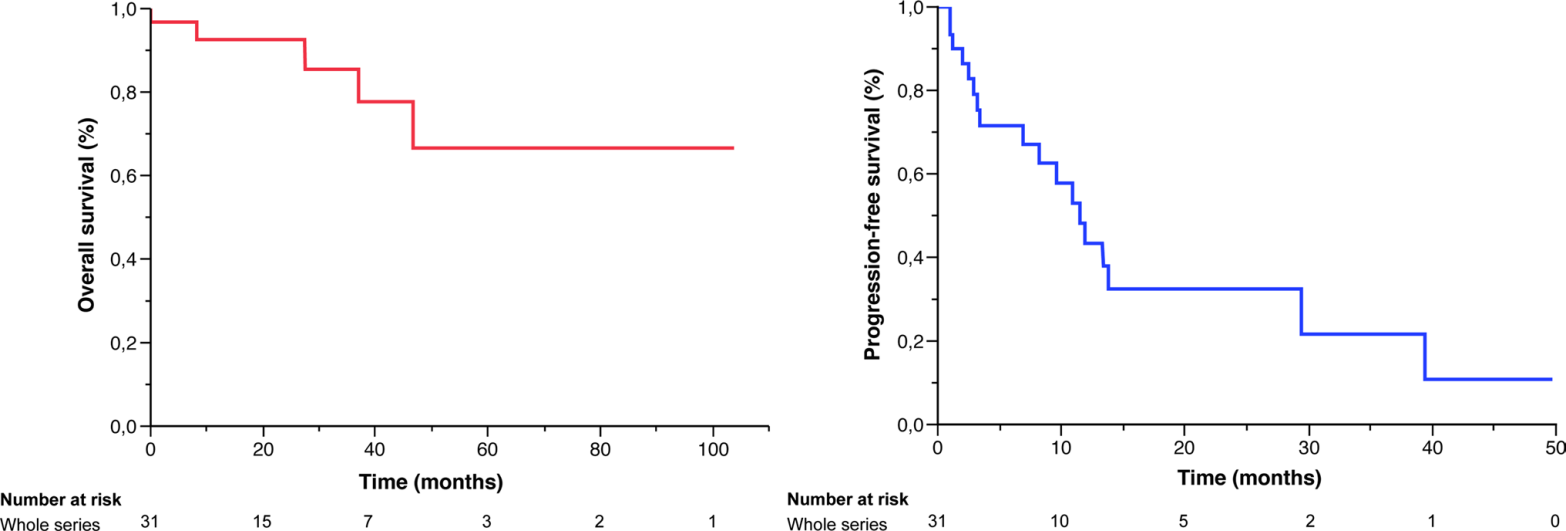
OS and PFS of 31 pediatric PAAF, in months (Kaplan Meier curves)

Outcome data were available for all patients included in the cohort, with a range 2 months–9 years and a mean follow-up period of 28.2 months (*σ* = 26.1 months). Eighteen (58%) patients had tumor recurrence, with a mean PFS of 16.6 months (median 11.5 months; CI 95% 6.9–29.4). Four patients died of their disease, and one during surgery, with a mean OS of 41.5 months (Fig. 4).

In a univariate analysis, using the Log-rank test, no clinical or histopathological features were significantly associated with a worse OS (*p* < 0.05). Multivariate analyses using the Cox proportional hazards model did not find any statistically significant predictors of OS (values *p* < 0.05).

Univariate PFS analyses using the log-rank test put forward the following clinical features as significantly associated with a shorter PFS: age at diagnosis ≤ 6 years (*p* = 0.014, CI 95% 6.9–29.4), midline location compared to posterior fossa (*p* = 0.0293), and partial tumor resection (*p* = 0.0357). Outcome analyses dependent on histopathological criteria were carried out. No significant outcome differences between cases of PA with anaplasia with necrosis *versus* no necrosis (*p* = 0.07), or with 4–6 mitoses versus 7 or more mitoses were evidenced (*p* = 0.857). For cases with available ASL values (*n* = 17), no significant association was found between ASL hyper-perfusion and PFS (*p* = 0.197).

**Fig. 5:**
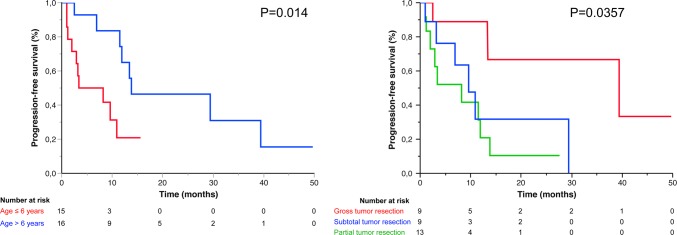
PFS according to age and extent of resection

Multivariate analyses performed with the Cox proportional hazards model revealed age at diagnosis ≤ 6 years (adjusted hazard ratio (HR) 7.08 [95% CI 2.03–31.05], *p* < 0.001) and partial surgical resection (adjusted HR 9.17, [95% CI 1.95–69], *p* < 0.015) as independent risk factors which significantly impacted progression free survival (Fig. 5).

An excel sheet detailing all the data from this publication can be found in supplementary Table 1, online resource. Methylation array data have been deposited in the ArrayExpress database at EMBL-EBI (www.ebi.ac.uk/arrayexpress) under accession number E-MTAB-8390.

## Discussion

We described the largest series of pediatric PAAF cases selected on the basis of the 2016 update of the WHO classification. Anaplasia in PA is defined by detailed criteria: ≥ 4 mitoses for 10 HPFs, with or without necrosis [3, 19, 26]. Two recent publications reinforced the existence of this entity, also called ANA PA or MC-AAP, based on a specific DNA methylation profile [3, 19, 26]. Indeed, this profile is different from the three conventional PA groups (PA PF, PA MID, PA SUP) [26]. This large study was based on 83 histopathologically defined PAAFs, mainly in adults (77/83) in infratentorial location (63/76) [26]. In the later study, mitotic cutoff of 4/10HPF was not reached in 61% of cases and MAPK alteration was not described in 25% of cases. The homozygous deletion of *CDKN2A/B* was found in 80% of cases, and loss of nuclear ATRX expression in 44% was considered important molecular distinctions, contrary to classical PA [26]. Our cohort of pediatric PAAF, based on strict morphological and molecular WHO criteria, did not show the typical methylation pattern observed in adults. Moreover, most of the cases exhibited the classical molecular criteria described in PA (*BRAF* fusion) and/or clustered with one of the three conventional PA methylation groups. t-SNE analysis reinforced that a majority of these tumors clustered with classical PA. These findings led us to consider the diagnosis of PAAF based on histological criteria in children with caution since these criteria can also be observed in bona fide PA.

When focusing on the rare pediatric cases of PAAF (summarized in Table 2), the histomolecular characteristics of our single MC-AAP (patient 27) were in fact not substantially different from the literature, although available detailed histomolecular data were less extensive than for adult MC-AAP [17, 21, 26–28]. Interestingly, PAAF and/or MC-AAP in children do not share the main characteristics described in their adult counterparts. In the 10 out of 22 published pediatric cases with a known MAPK alteration, very few presented with the classical *KIAA1549:BRAF* alteration (*n* = 3). One harbored a double MAPK and H3K27M mutation, whose biological significance in this setting is still uncertain, but which must clearly be distinguished from both single MAPK altered tumors and single H3K27M mutated tumors [18, 23]. In the remaining 9 cases, only four presented with a molecular profile similar to MC-AAP as defined in adults with a homozygous *CDKN2A/B* deletion, and only one also harbored a loss of ATRX protein expression. These findings have also been mentioned by Mistry et al. as alterations defining a subgroup of low-grade glioma with a tendency towards progression, including a single case with PA morphology [20]. However, in our cohort, no cases presented with a loss of ATRX and only one case showed a homozygous *CDKN2A* deletion, but was nonetheless classified as PA by DNA methylation profiling. Thus, the loss of ATRX and/or *CDKN2A* deletion, considered as hallmarks of MC-AAP in adults, does not seem to be strong indicators of MC-AAP in children. Overall, of the 7 pediatric cases (including the one from our cohort) with a diagnosis of MC-AAP confirmed by DNA methylation profiling, none presented with the most common anomalies described by Reinhardt et al.: *NF1* mutation, *CDKN2A* deletion, and ATRX loss. Therefore, we consider that the diagnostic criteria established for adult MC-AAP are not accurately applicable to the pediatric population. This reinforces the importance of comparing molecular anomalies of pediatric tumors between themselves and separately from adult tumors.

**Table 2:** 22 Published pediatric cases of PAAF, with or without DNA methylation profiling

Authors	Institutional histopathological diagnosis	DNA methylation profile	Location	Age at diagnosis (years)	Gender [M: male; F: female]	Death	OS (months)	Progression	PFS (months)	Mitotic count/10HPFs	Necrosis	MAPK alteration	*Homozygous* CDKN2A *deletion*	Loss of ATRX	PIK3/ATK alteration	Other alterations
Reinhardt 2018	Anaplastic pilocytic astrocytoma	MC AAP	Posterior fossa	0–18	F	NA	NA	NA	NA	NA	NA	*KIAA1549: BRAF*	1	1	NA	0
Reinhardt 2018	Anaplastic pilocytic astrocytoma	MC AAP	Spinal	0–18	F	NA	NA	NA	NA	NA	NA	*FGFR1* mut/fus	1	0	NA	0
Reinhardt 2018	Anaplastic pilocytic astrocytoma	MC AAP	Posterior fossa	0–18	M	NA	NA	NA	NA	NA	NA	*FGFR1* mut/fus	0	0	NA	MGMT promoter methylation
Reinhardt 2018	Anaplastic pilocytic astrocytoma	MC AAP	Posterior fossa	0–18	M	NA	NA	NA	NA	NA	NA	NOS	1	NA	NA	0
Reinhardt 2018	Anaplastic pilocytic astrocytoma	MC AAP	Posterior fossa	0–18	M	NA	NA	NA	NA	NA	NA	NOS	0	0	NA	0
Reinhardt 2018	Anaplastic pilocytic astrocytoma	MC AAP	Supratentorial	0–18	F	NA	NA	NA	NA	NA	NA	NOS	0	NA	NA	0
Lopéz 2018	Anaplastic trans-formation of PA	NA	Cerebellum	2	F	NA	NA	1	10	8	0	*NTRK2*	1	0	0	0
Rodriguez 2011 case 1 /2019 case 17	Anaplastic pilocytic astrocytoma	NA	Cerebellum	5	M	A	NA	NA	NA	≥ 4	0	*BRAF* duplication	0	0	pAKT/pS6 IHC	
Rodriguez 2011 case 4/2019 case 25	Anaplastic pilocytic astrocytoma	NA	R frontal lobe	11	M	A	NA	NA	NA	≥ 4	1	NEC	0	0	pAKT/pS6 IHC	
Rodriguez 2011 case 10/2019 case 22	Anaplastic pilocytic astrocytoma	NA	3rd ventricle	10	M	D	NA	NA	NA	≥ 4	1	NEC	0	1	pAKT/pS6 IHC	H3K27M
Rodriguez 2011 case 18	Anaplastic pilocytic astrocytoma	NA	Spinal	16	M	NA	NA	NA	NA	≥ 4	1	NEC	NA	NA	*PTEN* het del + pAKT IHC	
Rodriguez 2011 case 24/2019 case 26	Anaplastic pilocytic astrocytoma	NA	R occipital lobe	11	F	A	NA	NA	NA	≥ 4	0	NF1	NA	0	pS6 IHC	
Rodriguez 2011 case 25/2019 case 27	Anaplastic pilocytic astrocytoma	NA	Posterior fossa	14	M	D	NA	NA	NA	≥ 4	1	NOS	NA	0	NA	
Rodriguez 2019 case 1	PA with anaplasia	NA	Cerebellum	17	M	D	NA	NA	NA	≥ 5	1	NEC	NA	1	0	
Rodriguez 2019 case 3	PA with anaplasia	NA	Cerebellum	13	F	A	NA	NA	NA	≥ 5	1	NOS	NA	1	0	
Rodriguez 2019 case 4	PA with anaplasia	NA	Cerebellum	12	F	D	NA	NA	NA	≥ 5	1	NF1	NA	0	0	
Rodriguez 2019 case 11	PA with anaplasia	NA	3rd ventricle	12	M	A	NA	NA	NA	≥ 5	1	NEC	NA	0	0	
Rodriguez 2019 case 12	PA with anaplasia	NA	L hemisphere	3	M	A	NA	NA	NA	≥ 5	1	NEC	NA	0	0	
Rodriguez 2019 case 13	PA with anaplasia	NA	Posterior fossa	4	M	D	NA	NA	NA	≥ 5	1	*KRAS*	0	1	0	H3K27M
Olar 2019	PAAF	NA	Cerebellum	1.6	M	A	49	0	0	≥ 4	1	*KIAA1549: BRAF*	NA	1	loss of PTEN/ pS6 + IHC	
Olar 2019	PAAF	NA	Cerebellum, midline	12	F	A	47	0	0	≥ 4	1	NOS	NA	1	loss of PTEN/ pS6 + IHC	
Mistry 2015	PA undergoing trans-formation	NA	Midline	> 5	F	NA	NA	NA	NA	NA	NA	BRAF V600E	1	NA	NA	

*A* alive, *D* deceased, *het del* heterozygous deletion, *IHC* immunohistochemistry, *MC AAP* methylation class anaplastic astrocytoma with piloid features, *NA* not available, *NEC* not elsewhere classified, *NOS* not otherwise specified, *PA* pilocytic astrocytoma

Furthermore, it should be highlighted that the study on adult MC-AAP found somatic *NF1* mutations as the most frequent MAPK alteration, associated with *CDKN2A/B* and ATRX loss [26]. Interestingly, a study on gliomas arising in neurofibromatosis 1 (NF1) found the same frequent *CDKN2A* and ATRX genetic alterations in NF1 high-grade gliomas [5]. These cases were subjected to DNA methylation profiling and did not correspond to MC-AAP [5]. It would, therefore, be misleading to use only molecular criteria to define MC-AAP within or outside the setting of background NF1-associated tumor predisposition. The only case of clinical NF1 in our cohort (patient 31) presented with a loss of *CDKN2A/B*. In this circumstance, a germline *NF1* mutation followed by a somatic mutation lead to the loss of heterozygosity of the *NF1* gene, thus causing the development of glioma [5]. This strongly supports the cooperation between an *NF1* alteration and the loss of *CDKN2A/B* to bypass oncogene-induced senescence [9].

Few of the existing studies reported alterations of the PIK3/AKT pathway, with 3/7 tested cases presenting with a loss of PTEN, phospho-S6, and/or phospho-AKT expression (Table 2) [21, 27]. Within our cohort, loss of PTEN expression was described in 17% of PA, and our single MC-AAP showed monosomy at the *PTEN* locus by FISH analyses, but no homozygous deletion as previously described [21]. Immunohistochemistry for our cases revealed phospho-S6 loss in 3% of cases, conserved PTEN staining in 69% of cases, and partial or complete loss in 31% (17% and 14%, respectively). In PAAF, *PTEN* homozygous deletion was suggested to be associated with worse survival [21], but this was not confirmed by the present study.

Radiological characteristics of PAAF/MC-AAP have not yet been described. After central neuroradiological review, the tumors presented with an aspect compatible with classical PA. The single MC-AAP was slightly different, since it was the only hemispheric case, and suggested the differential MRI diagnoses of ependymoma, *RELA*-fusion positive or cortical PA. No ASL values were available for this case. However, it was noticed that ASL values were higher than previously described in classical PA [6].

Considering the clinical and prognostic aspects of our cohort, pediatric PAAF are not substantially different from classical PA. A large study on pediatric PA put forward patient age and extent of resection as the key prognostic factors, both of which were also the significant variables associated with PFS in our multivariate analyses [30]. Mitotic count above 4 per 10 HPF and the presence of necrosis were not of prognostic value among the cases analyzed in this study.

One study has suggested that PAAF in children had a better prognosis than in adults, but the DNA methylation class of these tumors is not known [29] and survival data on MC-AAP confirmed by DNA methylation profiling are not described in the literature (Table 2). The outcome for the only patient with MC-AAP seemed rather unfavorable, with an OS of 3.1 years following GTR and combined radiotherapy and chemotherapy. Further studies with extensive follow-up data are needed to define the prognostic significance of MC-AAP in children.

The large proportion of PAAF reclassified by DNA methylation profiling as classical PA suggests that the diagnoses to consider when faced with histopathological PA with elevated mitotic count with or without necrosis in children are firstly classical PA, and more rarely DLGNT, GBM *IDH*-WT, or MC-AAP.

The differential diagnosis of DLGNT may be complicated by its wide spectrum of histopathological and radiological features [7]. The molecular hallmarks of this tumoral entity are the same recurrent MAPK alterations as described in classical PA, associated with a loss of chromosome 1p [7]. The distinction from classical PA may be complicated due to the variety of possible MAPK alterations, tissular heterogeneity (major piloid component, absence of neuropil islands), and sub-clonal 1p deletion. In our case, 1p deletion was heterogeneous, not observed in all the tumoral components.

In our case of MC-AAP, the main differential diagnosis radiologically was ependymoma, *RELA*-fusion positive, and histopathologically was hemispheric GBM. The mitotic activity of this tumor was considerably more elevated than the aforementioned 4 mitoses described in PAAF (17 mitoses for 2.3 mm^2^), and it featured necrosis and microvascular proliferation. The CNP also revealed an amplification of *MDM2* gene, confirmed by immunohistochemistry, as previously described in GBM [13]. The 2 GBMs, *IDH*-WT in our series had 16 and 32 mitoses for 2.3mm^2^ and microvascular proliferation, with or without necrosis. The diagnosis of supratentorial pediatric GBM can in itself be complex if *IDH1/2* and histone gene mutations are not found [15].

The distinction is further complicated by the fact that the MC-AAP and GBMs, *IDH*-WT all harbored *FGFR1* alterations. Alterations of *FGFR1* have been described in the form of somatic mutations, gene fusions, and intragenic tyrosine kinase domain (TKD) duplications both in pediatric low-grade gliomas and glioblastomas [11, 25, 32]. The same hotspot *FGFR1* mutations have been described in PA and GBM alike, and were found in our cohort in both a classical PA (N546K) (patient 25) and the spinal GBM, *IDH*-WT (N546S and K656E) (patient 26) [11, 25]. Our MC-AAP harbored an *FGFR1* K687E mutation. The other GBM, *IDH*-WT (patient 28), harbored both an *FGFR1* TKD-duplication and an E454K *PIK3CA* mutation. Activation of the PIK3CA pathway is considered an important genetic event in the development of GBM and can be associated with MAPK pathway activation [2].

This reinforces the notion that isolated anomalies of *FGFR1* are not a diagnostic or grading argument in pediatric glioma, as they have been described in benign glioneuronal (dysembryoplastic neuroepithelial tumors, and Rosette-forming glioneuronal tumor), glial (PA) and malignant neoplasms alike (GBM, and diffuse midline glioma, H3 K27M-mutant) [8, 10]. Pediatric gliomas compatible with PAAF with a very high mitotic index (in our experience, more than 10 mitoses for 10 HPFs) should benefit from DNA methylation profiling, especially in the context of nonspecific *FGFR1* alterations. We suggest that in cases of pediatric tumors with piloid features and > 10 mitoses for 10 HPFs the diagnoses of GBM and MC-AAP can be considered, but should be confirmed by DNA methylation profiling.

Therefore, based on our cohort and on the few published pediatric cases, the histological and molecular criteria that define anaplasia in adult PA cannot be applied for diagnosis in children. In this setting specifically, these tumors correspond either to PA or various other diagnoses including DLGNT and GBM. As the specific molecular alterations (*CDKN2A* deletion and ATRX loss) described by Reinhardt et al*.* have not been found in this cohort, one can conclude that the MC-AAP is rare in children, justifying further cooperative studies to define this entity in the pediatric population through the integration of multiple layers of information.

## Electronic supplementary material

Below is the link to the electronic supplementary material.

Supplementary Table 1, online resource: detailed summary of all the information available in this paper. (XLSX 19 kb)

Supplementary material 2. (TIFF 12 mb)
