# Supplementary figures and images for: The histomolecular criteria established for adult anaplastic pilocytic astrocytoma are not applicable to the pediatric population
Source: Acta Neuropathol. 2019 Nov 1;139(2):287–303. doi: 10.1007/s00401-019-02088-8 (PMC6989446; doi:10.1007/s00401-019-02088-8)

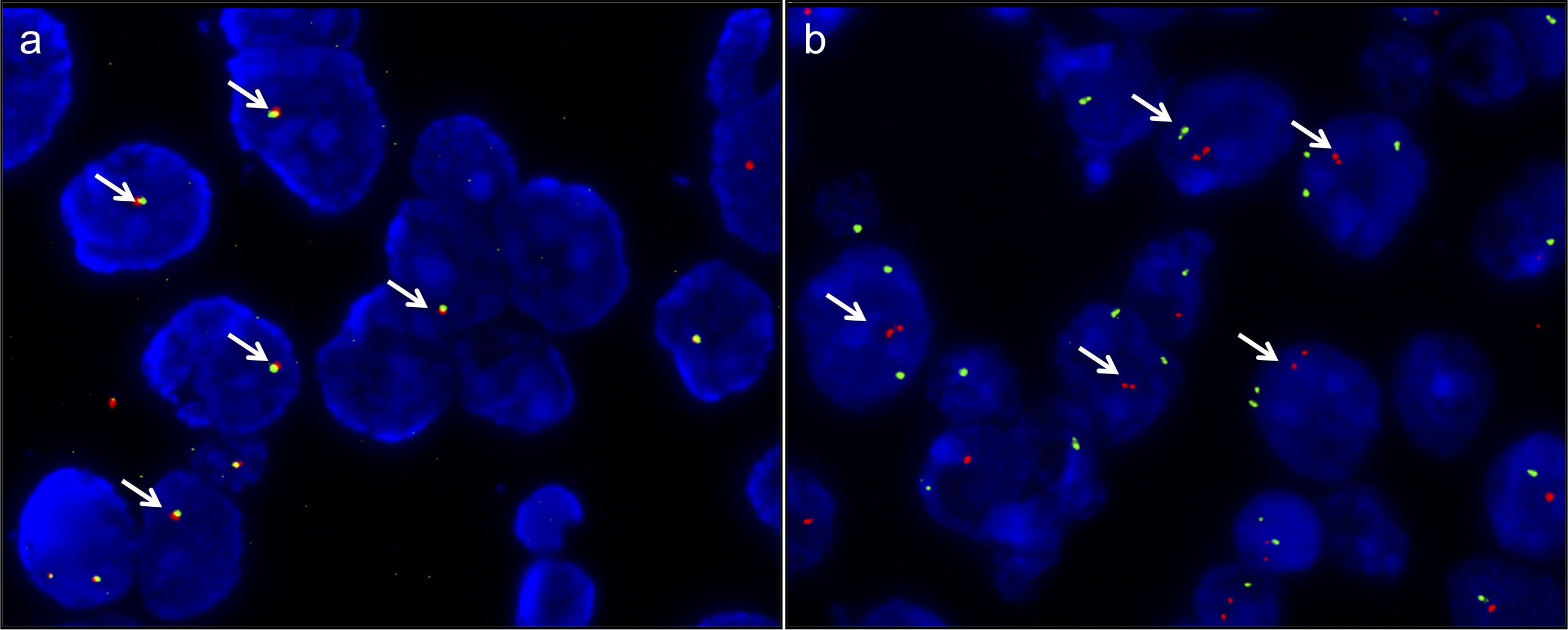

Supplement: Supplementary file 2 — Supplementary material 2. (TIFF 12 mb) [file 401_2019_2088_MOESM2_ESM.tiff]
